# Age-related cognitive impairment is associated with long-term neuroinflammation and oxidative stress in a mouse model of episodic systemic inflammation

**DOI:** 10.1186/s12974-018-1059-y

**Published:** 2018-01-30

**Authors:** Joana Costa d’Avila, Luciana Domett Siqueira, Aurélien Mazeraud, Estefania Pereira Azevedo, Debora Foguel, Hugo Caire Castro-Faria-Neto, Tarek Sharshar, Fabrice Chrétien, Fernando Augusto Bozza

**Affiliations:** 1 Laboratory of Immunopharmacology, Oswaldo Cruz Foundation (FIOCRUZ), Rio de Janeiro, Brazil; 2 National Institute of Infectious Diseases Evandro Chagas, Oswaldo Cruz Foundation (FIOCRUZ), Ministry of Health, Rio de Janeiro, Brazil; 3 D’Or Institute for Research and Education (IDOR), Rio de Janeiro, Brazil; 4 Human Histopathology and Animal Models, Pasteur Institute, Paris, France; 5 Institute of Medical Biochemistry Leopoldo DeMeis, Federal University of Rio de Janeiro, Rio de Janeiro, Brazil

**Keywords:** Aging, Microglia, Nox2, Sepsis, Brain, Cytokines

## Abstract

**Background:**

Microglia function is essential to maintain the brain homeostasis. Evidence shows that aged microglia are primed and show exaggerated response to acute inflammatory challenge. Systemic inflammation signals to the brain inducing changes that impact cognitive function. However, the mechanisms involved in age-related cognitive decline associated to episodic systemic inflammation are not completely understood. The aim of this study was to identify neuropathological features associated to age-related cognitive decline in a mouse model of episodic systemic inflammation.

**Methods:**

Young and aged Swiss mice were injected with low doses of LPS once a week for 6 weeks to induce episodic systemic inflammation. Sickness behavior, inflammatory markers, and neuroinflammation were assessed in different phases of systemic inflammation in young and aged mice. Behavior was evaluated long term after episodic systemic inflammation by open field, forced swimming, object recognition, and water maze tests.

**Results:**

Episodic systemic inflammation induced systemic inflammation and sickness behavior mainly in aged mice. Systemic inflammation induced depressive-like behavior in both young and aged mice. Memory and learning were significantly affected in aged mice that presented lower exploratory activity and deficits in episodic and spatial memories, compared to aged controls and to young after episodic systemic inflammation. Systemic inflammation induced acute microglia activation in young mice that returned to base levels long term after episodic systemic inflammation. Aged mice presented dystrophic microglia in the hippocampus and entorhinal cortex at basal level and did not change morphology in the acute response to SI. Regardless of their dystrophic microglia, aged mice produced higher levels of pro-inflammatory (IL-1β and IL-6) as well as pro-resolution (IL-10 and IL-4) cytokines in the brain. Also, higher levels of Nox2 expression, oxidized proteins and lower antioxidant defenses were found in the aged brains compared to the young after episodic systemic inflammation.

**Conclusions:**

Our data show that aged mice have increased susceptibility to episodic systemic inflammation. Aged mice that showed cognitive impairments also presented higher oxidative stress and abnormal production of cytokines in their brains. These results indicate that a neuroinflammation and oxidative stress are pathophysiological mechanisms of age-related cognitive impairments.

**Electronic supplementary material:**

The online version of this article (10.1186/s12974-018-1059-y) contains supplementary material, which is available to authorized users.

## Background

Aging is normally associated with a physiological decline that contributes to worse quality of life. The decline of aging is not always linear or consistent but can be periodic depending on the individual’s life style, genetic, and environmental factors [1–3]. The gradual accumulation of defective molecules and cells during aging contribute to disease progression in different ways. Evidence shows that systemic inflammation episodes, such as severe infections [4, 5], trauma, or major surgeries are associated with neurological alterations that can manifest acutely or long-term, increasing the risk of neurodegenerative diseases in these individuals [6–10].

Deregulated immunity in the elderly, or immunosenescence, contributes to their increased susceptibility to disease and impact mortality [11]. Aging involves the senescence of cells, organs, and systems and immunosenescence leads to defects in both innate and adaptive immune responses [12]. Microglia are the resident immune cells of the central nervous system, and their functions are also affected by age [13]. Microglia function is essential to maintain the brain homeostasis by their constant surveillance of the tissue, as well as their response to stressful insults, actively participating in neuroinflammation [14]. Recent evidence show that microglia release soluble factors, including cytokines and prostaglandins, which reciprocally influence and modulate neuronal function, shaping and maintaining the synaptic network in physiologic and pathophysiologic conditions [15, 16]. Neuroinflammation is associated with virtually all neurodegenerative diseases, psychiatric disorders such as depression and severe infectious diseases such as sepsis [17–19]. Activated microglia produce inflammatory mediators and reactive oxygen species that contribute to tissue injury and neurotoxicity by mechanisms that include oxidative stress and synapse remodeling that have been associated to cognitive impairments [20, 21].

Clinical and pre-clinical studies have shown that aged microglia are primed and present exacerbated response to acute inflammatory challenge [7, 22]. But the long-term consequences of systemic inflammation in aged mice have not been explored in episodic disease models. Our hypothesis is that episodic systemic inflammation, such as recurrent infections common in the elderly, could accelerate age-related cognitive decline due to an increased vulnerability of the aging brain. This study aimed to identify vulnerability factors and neuropathological features associated with age-related cognitive decline in a mouse model of episodic systemic inflammation.

## Methods

### Animals

Adult male Swiss mice (2 months old, 25 to 30 g) and aged male Swiss mice (12 months old, 40 to 45 g) were obtained from the Laboratory Animal Breeding Center (ICTB) of Oswaldo Cruz Foundation (FIOCRUZ). The animals were maintained on a 12:12-h light–dark cycle with free access to food and water. Aged mice were maintained one per cage over the course of experiments. All procedures were performed in accordance with international guidelines for use of Laboratory Animals and approved by the Institutional Animal Welfare Committee of FIOCRUZ (CEUA), Rio de Janeiro, Brazil.

### Experimental model

**Fig. 1:**
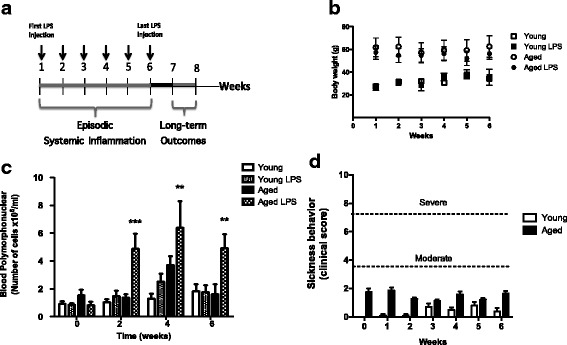
Experimental design. **a** The experimental model of episodic systemic inflammation (SI) was developed by six weekly intra-peritoneal injections of lipopolysaccharide (0.3 mg/kg). Long-term outcomes were evaluated 1 week after SI and include behavioral tests, blood and brain cytokine levels, and brain oxidative stress markers. **b** Body weight was not affected by LPS treatment. **c** Systemic inflammation was accompanied by monitoring peripheral blood neutrophils in the baseline (before the first LPS injection) and 24 h after the 2nd, the 4th, and the 6th LPS injections. Systemic inflammation was higher in aged mice (aged LPS) compared with young mice submitted to the same treatment and with counts at baseline. **d** Sickness behavior of young and aged mice was evaluated 24 h after each weekly LPS injection (1st, 2nd, 3rd, 4th, 5th, and 6th LPS injections). Mild sickness behavior was significantly increased in aged mice. Data were plotted as means ± SEM (*n* = 10–15 per group, from three independent experiments). Statistical analysis with two-way ANOVA and Bonferroni posttest (*p < 0.05, **p < 0.01, ***p < 0.001)

Young and aged mice were randomly divided into two groups (*n* = 10 per group for each experiment): naive controls or episodic systemic inflammation (SI). Episodic systemic inflammation (SI) was produced by weekly injections of a lipopolysaccharide solution (LPS from *Escherichia coli* 0111:B4, Sigma-Aldrich, Inc.) prepared in physiological saline (sterile 0.9% NaCl). Intraperitoneal injections of LPS (0.33 mg/kg) were made in 100 μL volume using 1 mL syringes with 30G × 12.7 mm needles; the solution was sonicated for 5 min before injections. All injections were given at 12:00 noon (± 1 h), once a week for 6 weeks to produce an episodic systemic inflammation without inducing tolerance to LPS due to the 1 week interval between injections [23]. Control mice were manipulated the same way as LPS-injected animals, but were not injected with vehicle to avoid any lesion that could induce a local inflammation (naive controls). Sickness behavior and systemic inflammation were assessed in different phases of episodic systemic inflammation in young and aged mice by a researcher blinded to experimental groups (control or SI) (Fig. 1). To monitor systemic inflammation during the evolution of SI, we evaluated white blood cells (WBC) in the peripheral blood at several time points after LPS injections. One drop of blood was collected from the mouse-tail, made free of red blood cells with Türk solution and the total number of WBC was manually counted in a Neubauer chamber. Peripheral blood differential count was made in blood smears, air dried, and stained with panoptic technique. The percentages of individual morphological forms of WBC were established and absolute number of blood polymorphonuclear granulocytes (neutrophils) was calculated by absolute cell count/mL = total WBC/mL × % of the cell on the differential count. Long-term outcomes (behavioral, biochemical, and immunohystochemical studies) were evaluated 1 to 2 weeks after the last LPS injection (Fig. 1c). Aged mice used in this study were middle-aged that presented signs of senescence, such as lipofuscin deposits [24, 25] (Additional file 1: Figure S1). This experimental model overall did not induce severe systemic inflammation or mortality. However, a few aged mice died after SI (less than 10% mortality). Some aged mice presented tumors that were detected after euthanasia, and these animals were excluded from the study. Aged mice that developed severe symptoms were also excluded from the study and euthanized (less than 5%).

### Behavioral tests

All the procedures used in this study were designed to minimize the potential discomfort during behavioral tests. Clinical manifestations of sickness behavior were evaluated 24 h after each weekly LPS injection, when mice were scored as being mildly, moderately, or severely affected as described previously with some modifications [7, 26, 27]. Briefly, the scoring assessed mice appearance (piloerection, bloated abdomen, sunken eyes), alertness (interest in the environment, moving away from experimenter hand, locomotion), grip strength (time holding to a bar 30 cm high with fore paws), and body temperature (measured with an infrared thermometer) (Fig. 1d). After SI, 1 week after the last LPS injection, all groups of mice were submitted to behavior tests. Depressive-like behavior was evaluated with the forced swimming test. Mice were placed in a transparent cylindrical tank filled with temperature-controlled tap water (25 °C). Mice were hold by its tail, gently and slowly placed in the water, and their immobility time was recorded for 3 min. The lack of escape-related mobility behavior is measured by the immobility time and indicates depressive-like behavior.

Episodic-like memory of mice was assessed with the object recognition test. This type of memory is required to the natural exploratory abilities of rodents exposed to a new environment and involves medial temporal lobe neural circuits (hippocampus and entorhinal cortex). The object recognition test was performed according to a methodology previously described [28] in a square wooden open-field apparatus (40 × 40 × 40 cm) and consisted of three phases: habituation, familiarization, and test phase. In the habituation phase each animal was allowed to freely explore the open-field arena in the absence of objects for 10 min. The familiarization phase was performed 24 h after habituation and consisted of a 5 min exploration of identical objects in the arena. The test was made 3 h after familiarization phase and consisted of 5 min exploration of two different objects, one familiar and a substituted novel object. The novel object had different color, shape, and size that allowed recognizing it as novelty. The time each animal explored the familiar and the novel object was filmed, and an observer blinded to experimental conditions analyzed the videos. Exploration was defined as the animal directing its nose within 2 cm of the object while looking at, sniffing, or touching it. The arena and objects were cleaned with a 10% ethanol solution between trials to ensure that behavior of animals was not guided by odor cues. An exclusion criterion based on a minimal level of object exploration was used to exclude animals with naturally low levels of spontaneous exploration (novel + familiar exploration time < 10 s). Animals that explored the objects for less than 10 s were excluded from the study. The object recognition index was calculated as time for the novel object − time for the familiar object/total exploration time.

The Morris water maze test was performed as previously described [18] 1 week after the last injection of LPS, to evaluate mice spatial memory. This type of memory, which also involves the medial temporal lobe, permits the formation of a cognitive map of the external space to which the animal is located and through which allow it better interaction with the environment [29]. The water maze consisted of a circular tank with a diameter of 1.04 m and height of 55 cm, located inside a room with some visual cues fixed in the walls. The tank was filled with water and divided into four quadrants, and in each quadrant, a transparent acrylic platform (13 × 30 × 13 cm) submerged 1 cm from the water surface was placed. The experiment was recorded in a Samsung camera. The animals were subjected to daily training sessions for 4 consecutive days to find the platform. If the animal did not find the platform after 60 s, it would be conducted manually to it after 60 s. During all training sessions, the time each mouse required to find the platform (latency) was registered. In the fifth and final day of experiment, the test session was held, in which the platform was removed and mice were challenge to find the platform quadrant. The time mice spent in the platform quadrant in the test session, velocities of swimming, and latency time in the training sections were analyzed with the ANY-maze behavioral tracking software.

### Microglia morphology study

Microglia morphological studies were carried out at 4 h after the first LPS injection (acute SI) and 1 week after the last LPS injection (long-term SI) in the hippocampus of young and aged mice. Initially, microglia activation was evaluated by counting the total number of Iba1-positive cells and the number of Iba1-positive cells with activated morphology per field and expressed as percentage of activated microglia. Microglia were classified as activated when cells presented shortening of processes and increase in cell body size (hypertrophy) [30, 31]. A researcher blinded to experimental conditions performed this counting using the ImageJ Cell Counter plugin; at least four fields were counted per animal. A second study was developed to assess the acute morphological changes of microglia in the entorhinal cortex in response to SI (4 h after the last LPS injection). Microglia were considered dystrophic when abnormalities in cytoplasmic structure were observed, such as formation of cytoplasmic spheroids, gnarling, beading, and fragmentation, and a distinct loss of fine branches (deramification). As it is rare to see all of these dystrophic changes occurring in a single cell, most dystrophic microglia display just one or two of these characteristics [31]. The criteria used to classify dystrophic microglia were the presence of at least one of these features. For immunohistochemistry, the brains were dissected and immediately immersed in 4% paraformaldehyde for 3 days at 4 °C. After fixation brains were crioprotected in 20% sucrose for 2 days, frozen in dry ice, and stored at − 80 °C. Coronal sections (40 μm) were obtained in a cryostat (Leica Microsystems, Germany), washed with phosphate buffer saline at pH 7.4, and blocked for 2 h with 1% BSA, 2% normal goat serum and 0.3% Triton X-100 blocking solution at room temperature. Sections were incubated overnight at 4 °C free floating in primary antibody solution. For microglia studies, a rabbit IgG anti-Iba1 (1:400 dilution; catalog number 019-19741 from Wako Chemicals USA, Inc.) primary antibody in blocking solution was used. For oxidative stress studies, a rabbit IgG anti-HNE (1:400 dilution; catalog number HNE11-S from Alpha Diagnostic International Inc., Texas, USA) primary antibody in blocking solution was used. After primary antibody incubation, the sections were washed with PBS and incubated with the secondary antibody Alexa Fluor 488 goat anti-rabbit IgG (1:1000 dilution; catalog number A-11008 from Molecular Probes, Eugene, OR, USA) for 2 h at room temperature and washed with PBS. Sections were mounted in Vectashield Antifade Mounting Medium with DAPI (for nuclei counterstaining). No reactivity was observed in brain sections when the primary antibody was absent.

### Automated quantification of microglia morphology

Images of microglia from the hippocampus and entorhinal cortex were obtained using a Leica confocal microscope (Leica Microsystems, Germany) or a Zeiss confocal microscope (Carl Zeiss Microscopy, Germany) with Z stack and 3D-reconstruction. Confocal image stacks were used for microglial morphological studies. For quantification of morphological changes, microglial cells were automatedly segmented and separated from their neighboring ones after identification of their cell bodies. First, contrast was optimized using the local contrast enhancer plugin from Fiji, and an automatic threshold was determined with the Li algorithm. Microglial masks were automatically simplified using “open” and “close” functions. Individual cell bodies were obtained through the Iba1 channel, after local contrast optimization, automatic local threshold was determined with the Phansalkar algorithm set with a radius of 20 pixels. Then, a tridimensional watershed was performed (mcib3d suite). Only individual microglial cells were furthered considered for analysis. Cells were skeletonized using 3D skeletonize plugin and labeled for slab, junctions, or end of branches. Conformational Sholl analysis was performed through concentric envelopes computed with a 1 μm radius step starting from the cell body. Densities of slabs, junctions, or end of branches were then analyzed iteration after iteration to compute the number of branches as a function of the distance to cell body. Skeleton was also analyzed using the skeleton analysis algorithm, and the longest path was considered for each cell. The area under the curve was computed with GraphPad Prism software for each microglial cell and microglial cells (3–7 per samples) were pooled for each condition and compared using a Kruskall Wallis test.

### Cytokine measurements

Brain tissue and serum were frozen at − 80 °C for posterior analysis of cytokines (IL-1β, IL-6, IL-4, and IL-10), via enzyme-linked immunosorbent assay (ELISA, DuoSet – R&D Systems, USA), in accordance with the manufacturer’s instructions. The brains were homogenized in phosphate-buffered saline containing 0.1% Triton and protease inhibitor cocktail (Roche, Germany) and then centrifuged (12,000 × *g* at 4 °C for 20 min). The supernatant was collected and protein content evaluated using the Bicinchoninic Acid (BCA) Assay. Data were calculated as picogram/milligram protein of brain tissue or picogram/milliliter of serum and expressed as fold changes in the graphs.

### Western blotting for 4-hydroxynonenal

Oxidative stress in the brain tissue was quantified by immunodetection of 4-hydroxynonenal (4-HNE), a product and mediator of oxidative stress that binds covalently to proteins [32]. Mice were deeply anesthetized with isoflurane and transcardially perfused with 0.9% NaCl saline, and their brains were dissected and immediately frozen in dry ice. The tissue was homogenized 50 mM Tris, pH 8.0 containing 150 mM sodium chloride, 1 mM EDTA, 1% Triton X-100, 0.5% sodium deoxycholate, 0.1% SDS, protease inhibitor cocktail (Complete Mini Protease Inhibitor Cocktail; Roche Diagnostics, IN, USA), and phosphatase inhibitor sodium orthovanadate. Homogenates were incubated for 30 min on ice, sonicated for 3 min, and centrifuged at 10.000 × *g* at 4 °C. The supernatant was collected, and the protein concentration was measured using a BCA Protein Assay (Thermo Fisher Scientific, Waltham, MA, USA). For Western blotting, 50 μg of protein samples were separated on polyacrylamide 4–20% gradient gels (Mini-Protean TGX precast Gels®-BioRad, Hercules, CA, USA), and proteins were transferred to PVDF Immobillon-FL membranes (Millipore, MA, USA). Membranes were blocked with Odyssey Blocking Buffer reagent (Li-Cor Biosciences, Lincoln, NE, USA) and incubated overnight with a primary antibody mix of a mouse IgG anti-4-hidroxi-2-nonenal (1:500, Abcam, Cambridge, MA, USA) and a goat IgG anti-actin (1:5000, Santa Cruz, CA, USA). After washing with PBS, membranes were incubated with IRDye secondary antibodies (Li-Cor Biosciences) for 30 min. Immunoreactivity was visualized with Odyssey Infrared Imaging System (Li-Cor Biosciences, Lincoln, NE, USA). Band intensities were calculated using the Image Studio Software. 4-HNE-positive bands consisted of a smear with stronger bands between 50 and 150 kDa that were selected for quantification. 4-HNE signals from each lane were normalized to corresponding actin band signals.

### Real-time PCR

The brains were collected and directly homogenized in 1 ml of TRIzol® Reagent (Thermo Fisher Scientific Inc.), and total RNA was isolated following the manufacturer’s suggested protocol. RNA concentration was quantified using the NanoDrop 2000 Spectrophotometer (Thermo Scientific), and cDNA was synthesized according to manufacturer’s instructions (SuperScript First-Strand Synthesis System for RT-PCR, Invitrogen) and was stored at − 20 °C. Transcripts obtained from the reverse transcriptase reaction were quantified by quantitative fluorogenic real-time PCR using SYBR® green. The real-time PCR amplification was performed, recorded, and analyzed by a 7500 Real-Time PCR System (Applied Biosystems). The specificity of the SYBR® green assay was confirmed by melting-point analysis. The primers used were *Sod1* (left 5′ to 3′-GGA CCT CAT TTT AAT CCT CAC, right 5′ to 3′-TGC CCA GGT CTC CAA CAT G), *Cat* (left 5′ to 3′-AGA GAG CGG ATT CCT GAG AGA, right 5′ to 3′-ACC TTT CCC TTG GAG TAT CTG), *Sod2* (left 5′ to 3′-CAG ACC TGC CTT ACG ACT ATG, right 5′ to 3′-CTC GGT GGC GTT GAG ATT G), *Nox2* (left 5′ to 3′-GAC CAT TGC AAG TAG ACA CC, right 5′ to 3′-AAA TGA AGT GGA CTC CAC GC), and *Hprt* (left 5′ to 3′-AGG ACC TCT CGA AGT GTT GG, right 5′ to 3′-TTG CAG ATT CAA CTT GCG CT) at 300 nM concentration. Target gene expression was normalized by the *Hprt* gene. The levels of gene expression were calculated by the ΔCt method for quantification, and the results were expressed as 2^ΔCt^ [33].

### Statistical analysis

Statistical analyses and graphs were generated using GraphPad Prism version 5.0 (GraphPad Software). All results are presented as mean and standard errors of means (SEM). The data were analyzed using two-way ANOVA to determine how the two factors, age and SI, affected responses, with Bonferroni posttest for multiple comparisons, and Kruskal-Wallis with Dunn’s multiple comparisons test for non-parametric analysis, unless otherwise noted. Two-tailed univariate *t* test was also used, when appropriate. Statistical significance is shown on the graphs (*p < 0.05, **p < 0.01, ***p < 0.001). Statistical tests used for each data set are indicated in the figure legends.

## Results

### Aged mice are more vulnerable to cognitive impairments after episodic systemic inflammation

**Fig. 2:**
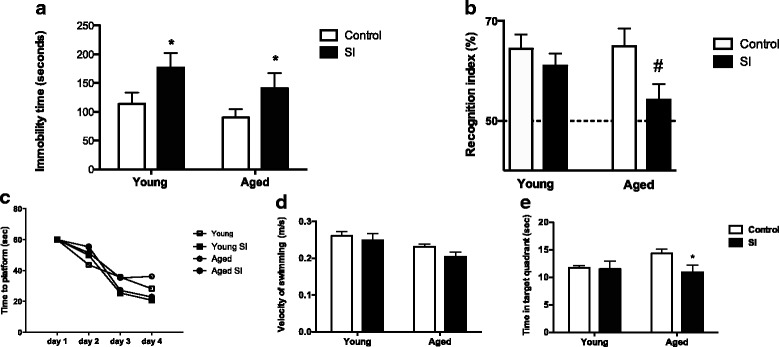
Aged mice are more susceptible to memory impairments after SI. **a** SI induced depressive-like behavior in both young and aged mice (*n* = 10 per group, from two independent experiments). **b** Object recognition test showed that SI affected short-term memory of aged mice only (aged SI). Statistical analysis two-tail univariate *T* test (*N* = 14–24 per group, from three independent experiments; # not statistically different from 50%). **c** Learning curves of mice for hidden platform water maze test were not significantly altered by age or SI. **d** Swimming velocity on the test day was not significantly affected by age or by SI. **e** Time in the target platform was significantly decreased in aged mice after SI on day 5. Each test was performed at least three times with different sets of mice. Figures show a representative water maze experiment. Data were plotted as means ± SEM (*N* = 10 to 13 per group). Statistical analysis with two-way ANOVA and Bonferroni posttest (*P < 0.05)

The long-term consequences of episodic systemic inflammation in the aging brain are poorly understood. Here, we developed a relevant pre-clinical model to investigate the molecular mechanisms of cognitive impairments associated with aging and episodic systemic inflammation (SI). The SI model consisted of weekly injections of LPS that induced mild symptoms of sickness behavior and higher systemic inflammatory response mainly in aged mice (Fig. 1). The behavior studies showed that SI effect differs depending on age and the cognitive domain tested. Depressive-like behavior was observed after SI in both groups of mice, young and aged (Fig. 2a). Cognitive domains such as memory and learning were affected only in aged mice. Young mice did not present deficits in the memory tests even after SI. However, aged mice were more susceptible to short-term memory impairments after SI, as shown by their poor performance in the object recognition test (Fig. 2b). All groups of mice were capable to swim and learn where the hidden platform was to perform the water maze test (Fig. 2c). Velocity of swimming was also similar between groups (Fig. 2d). Young controls and young SI had similar performances in the water maze test. However, aged SI mice spent less time in the target quadrant indicating they had compromised spatial memory compared to aged controls (Fig. 2e).

### Aged microglia are dystrophic and resistant to modulation

**Fig. 3:**
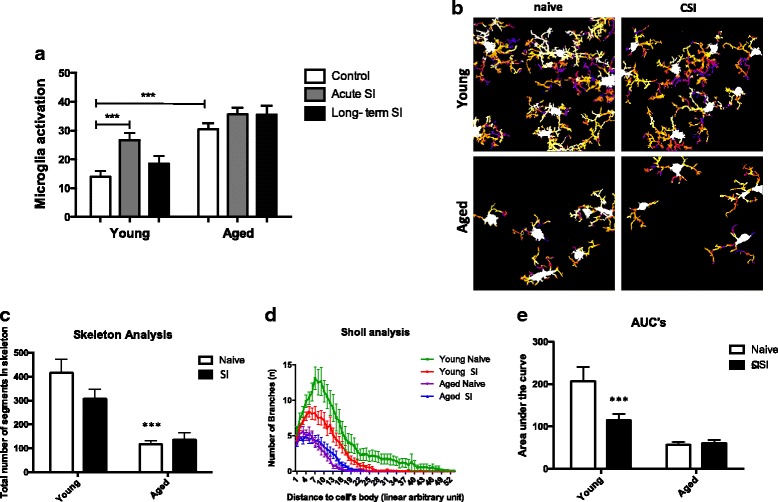
Sustained microglia activation in the aged brain. **a** Activated microglia were quantified throughout the hippocampus of mice and expressed as percentage of hypertrophic microglia over total number of Iba-1 positive cells (***P < 0.001). **b** Microglia masks from the entorhinal cortex of young and aged mice, naïve controls, and after SI. Microglial cell bodies are shown in white, and every circular envelope has a random color. **c** Total number of microglia skeleton segments are decreased by age (***p < 0.0001). **d** Sholl analysis of the number of branches plotted as a distance from cell body (*n* = 17 to 33, microglial cells per experimental group). **e** Area under the curve of the Sholl curves depicted in D showed a lower number of branches along the microglial cell in young naive vs young SI (207 ± 34 vs 115 ± 13, ***p < 0.001) but no difference between aged naive and aged SI (57 ± 7 vs 60 ± 8, *p* = 0.89). Statistical analysis with Kruskall Wallis test and Holm-Sidak’s multiple comparisons test. Data were plotted as means ± SEM

Immunohistochemistry analysis of Iba-1-positive cells allowed us to observe microglial morphology and the amount of activated microglia in the hippocampus of young and aged mice at different stages of SI to evaluate changes that were related to age or induced by SI. The percentage of activated microglia after SI was higher in young mice acutely and returned to basal levels long term after SI, while aged mice presented high amounts of activated microglia at the basal level that remained elevated long term after SI (Fig. 3a). We then studied the morphological features of microglia in higher magnification and in a quantitative way in the entorhinal cortex, a brain region that works as a major hub in a widespread network for memory and navigation of mice [34]. Young mice showed morphological features of surveillant microglia, long processes full of branches, whereas aged microglia were morphologically compatible with dystrophic microglia (Fig. 3b). Quantitative analysis of microglia morphological changes showed interesting differences between groups. Total number of microglia skeleton segments was lower in the aged mice (young 415 ± 57 vs aged 118 ± 13, *p* < 0.0001) whereas young SI showed a trend towards a diminution of microglial cell ramification number (Fig. 3c). Acute microglia response to episodic SI in maximal number of branches was higher in young mice (13.1 naive and 8.4 SI) than in the aged (5.6 naive and 4.9 SI), and SI had few effects on already poorly ramified microglial cell in aged animals (Fig. 3d). Sholl analysis showed a decrease in the number of branches along the microglial cell in response to SI in the young mice (young naïve 207 ± 34 vs young SI 115 ± 13, ***p < 0.001) but not between aged naive and aged SI (57 ± 7 vs 60 ± 8, *p* = 0.89) (Fig. 3e). Young microglia responded to SI by retracting their processes and decreasing the number of branches from their cell bodies. However, aged microglia did not present overt morphological changes upon SI challenge, being apparently resistant to modulation by SI (Fig. 3c–e).

### Sustained neuroinflammation and oxidative stress in the aged brain after episodic systemic inflammation

**Fig. 4:**
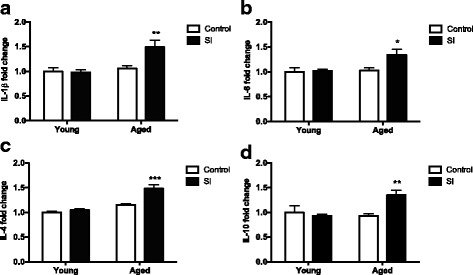
Brain cytokines levels long term after episodic inflammation. Brain cytokines IL-1ß (**a**), IL-6 (**b**), IL-4 (**c**), and IL-10 (**d**) were measured in young and aged mice naïve (control) and after episodic systemic inflammation (SI). All cytokines measured were significantly elevated in the brains of aged mice after SI. Data were plotted as means ± SEM (*N* = 8 per group, samples collected from 3 independent experiments). Statistical analysis with two-way ANOVA with Bonferroni posttest (*P < 0.05, **P < 0.01, ***P < 0.001)

Microglia are major regulators of neuroinflammation and produce most of the inflammatory mediators in the brain, such as cytokines and reactive oxygen species. The levels of cytokines IL-1β, IL-6, IL-4, and IL-10 were measured in the brains of young and aged mice after SI. Aged mice presented higher levels of both pro-inflammatory IL-1β and IL-6 and pro-resolution IL-4 and IL-10 cytokines in the brain compared to young long term after SI (Fig. 4).

**Fig. 5:**
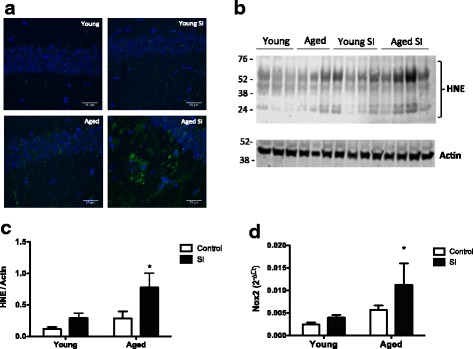
Oxidative stress is associated with increased Nox2 expression in the aged brains after episodic inflammation. **a** Confocal images showing increased 4-hydroxynonenal (HNE) immunostaining in the hippocampus of aged mice after SI. Scale bar = 25 μm. **b** Western blots for HNE protein adducts show increased content of oxidized protein in the brains of aged mice after SI. Molecular weights shown in kilodaltons (KDa). **c** Quantification of HNE western blot bands intensity, normalized by actin bands. **d** Real-time PCR analysis of gene expression was carried out with whole brain mRNA from young and aged mice, naïve (control), and long term after episodic systemic inflammation (SI). Data were plotted as means ± SEM (*N* = 4–5 per group). Statistical analysis with Mann Whitney test (*P < 0.05 relative to young controls)

**Fig. 6:**
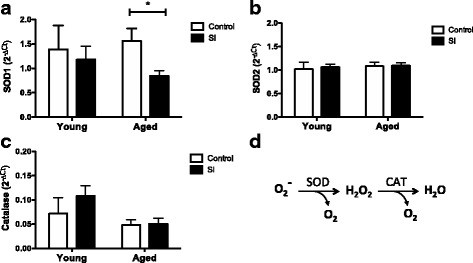
Brain antioxidant enzymes are downregulated by age and episodic inflammation. Real-time PCR analysis of gene expression was carried out with whole brain mRNA from young and aged mice, naïve (control) or after episodic systemic inflammation (SI). **a** SOD1 gene expression is significantly decreased in the brain of aged mice after SI. **b** SOD2 gene expression is not altered by age or SI. **c** Catalase gene expression is not altered by SI. Data were plotted as means ± SEM (*N* = 5 to 7 per group). **d** Chemical reactions of superoxide dismutase (SOD) and catalase (CAT) converting anion superoxide (O_2_^−^) into hydrogen peroxide (H_2_O_2_), oxygen (O_2_), and water (H_2_O). Statistical analysis with Mann Whitney test (*P < 0.05)

The hippocampus is a critical region for learning and memory processes in the brain, and it is especially susceptible to oxidative stress. We observed increased immunostaining for 4-HNE in the hippocampal CA1 region of aged mice after SI compared to the other experimental groups (Fig. 5a). Accordingly, higher concentrations of 4-HNE adducts in brain protein extracts from aged mice after SI, compared with the naïve controls (Fig. 5b, c). The oxidative stress observed in the aged brains long term after SI was temporally associated with an increased expression of Nox2 in the brains of these mice (Fig. 5c, d). We then measured the gene expression of major antioxidant enzymes superoxide dismutase 1 and 2 (*Sod 1 and Sod2*) and catalase (*Cat*) in the brains of young and aged mice, naïve, and long term after SI. Aged mice presented lower expression of *Sod1* after SI (Fig. 6a) and no differences in the expression of *Sod2* (Fig. 6b). Catalase expression was not affected by SI (Fig. 6c).

## Discussion

In the present study, we showed that episodic systemic inflammation (SI) induced age-dependent cognitive impairments that were absent in young submitted to the same treatment. Microglia from aged mice presented morphological features compatible with decreased surveillance. Moreover, SI induced an increase in the expression of Nox2 and a deficitary expression of SOD1 that may be responsible for the oxidative damage observed in the brains of aged mice. The memory impairments observed in the aged mice were associated with increased concentrations of inflammatory cytokines and oxidative stress in the brain.

Our experimental model resembles in many ways the neurological, inflammatory, and behavioral correlates of human aging. Human aging is characterized by low-grade, chronic, systemic inflammation in aging, in the absence of overt infection and is a highly significant risk factor for both morbidity and mortality in the elderly people [2]. Our data show that episodic peripheral injections of low-doses of LPS-generated exacerbated systemic inflammation and sickness behavior only in aged mice (Fig. 1). Behavior studies showed that cognitive domains such as memory and learning were particularly impaired in aged mice after SI, while depressive-like behavior was induced in both young and aged mice (Fig. 2). These results indicate that SI predispose mice to depressive behavior and that aged mice are more vulnerable than young to memory impairments, suggesting that brain regions involved with this type of behavior must be affected by SI in the aged mice, namely the medial temporal lobe region (hippocampus and entorhinal cortex).

As the resident immune cells of the brain, microglia are major regulators of inflammatory response in the central nervous system [35]. There is a close association between microglia morphology and the activation level of microglial cells under inflammatory stimuli. In homeostasis, non-stimulated, surveillant microglia present highly branched processes. Stimulated or primed microglia present increased cell body and less branched processes [35]. We studied microglia morphology in the hippocampus and the entorhinal cortex. Young naïve mice present highly ramified microglia, a morphological feature necessary for their constant surveying of brain parenchyma. Quantitative analysis of microglia morphological changes in response to LPS challenge after SI revealed that young microglia responded to LPS by retracting their processes and decreasing their number of branches, a morphological change compatible with activation and effective response to an inflammatory challenge. However, aged microglia ramifications were less branched and had shorter overall process lengths than those of young microglia, morphological alterations consistent with dystrophic microglia (Fig. 3). Microglia of the aged brain are marked by dystrophic morphology, characterized by deramification, cytoplasmic fragmentation, and shortening of cellular processes, even in the absence of frank pathology [31]. Another important aspect of aged microglia is an elevated inflammatory profile associated with a “sensitized” or “primed” phenotype. Mounting evidence points to a causal link between the primed profile of the aged brain and vulnerability to secondary insults, including infections and psychological stress [13, 22, 31, 35]. We showed that aged mice produced higher levels of cytokines in the brain long term after SI, compared to young mice after SI (Fig. 4), providing evidence of the presence of primed microglia in the brains of aged mice. Microglia deregulation by aging has been described elsewhere [13, 22, 36], and our experimental model corroborates this theory, providing evidence of the association between microglia deregulation and a deficitary brain function in aged animals induced by episodic systemic inflammation.

Episodic stimulation of the immune system is thought to be the most important cause of immunosenescence, and evidence shows that repetitive infections or “sterile” systemic inflammatory conditions accelerate age-associated pathologies [2, 37]. One aspect of repetitive immune stimulation with endotoxin is the development of tolerance. Endotoxin tolerance is defined as the reduced capacity of an immune cell to respond to subsequent LPS challenge after an initial exposure to this stimulus [23]. Behavioral and systemic consequences of repetitive inflammatory challenge have been investigated in different models of peripheral LPS challenge. In one study, repetitive LPS administration to adult rats did not change blood cytokine levels, indicating they developed endotoxin tolerance [23, 38]. Our experimental model did not induce endotoxin tolerance because we challenged mice with peripheral LPS at 1 week intervals, and the measurements of blood cytokines showed significant differences after the episodic treatment with LPS (Additional file 2: Figure S2). By the end of LPS treatment, aged mice presented a significant decrease in blood IL-4 (Additional file 3: Figure S3) indicating that Th2 and M2 phenotypes must be deficient in aged mice after SI [39, 40]. Different from the periphery, brain cytokine levels were higher in aged mice, both pro-inflammatory and pro-resolution cytokines (Fig. 4). These results suggest that increased pro-inflammatory cytokines IL-1ß and IL-6 in the brain are a consequence of primed microglia in the aged mice.

Inflammation is a protective response of our body that occurs in response to an insult. In the case of infection, the immune system is activated to identify the foreign agent and neutralize it. Eventually, the injurious stimulus overwhelms the protective effect, and inflammation may become self-perpetuating, contributing to tissue damage, which is the case of pathologic aging. Several studies have investigated how acute systemic inflammation, such as infections or surgical manipulation impact cognitive function in aging [41–46]. Aged rats challenged with *E. coli* infection showed prolonged elevations of IL-1ß when compared to young adults [47]. Similar results were obtained by Godbout and collaborators [7] with BALB/c mouse challenged with peripheral injection of LPS resulting in both exaggerated and prolonged elevations in IL-1ß and IL-6 in aged animals. Both studies [7, 47] demonstrated an exaggerated acute cytokine response of aged mice, more important in the brain than in the periphery. In our study, we aimed to clarify the neuropathology aspects long term after episodic systemic inflammation, a condition that is extremely common in the elderly and not very much explored in the experimental setting. Inflammatory response involves pro-inflammatory as well as pro-resolution cytokines. Under normal conditions, tissue-resident cells detect the damaging insult and alarm circulating leukocytes to migrate to the inflamed tissue to deal with the inflammatory insult. The clearance of apoptotic cells and restoring of tissue homeostasis require complex resolution processes that involve resolution-phase macrophages and pro-resolving immune mediators [48]. Episodic inflammatory conditions may be a result of defective resolution of inflammation. In our experimental model of episodic inflammation, aged mice produced higher concentrations of both pro-inflammatory and pro-resolution cytokines in the brain long term after SI, compared to young mice (Fig. 4). The simultaneous elevation of both pro-inflammatory and anti-inflammatory cytokines produced by activated microglia [49, 50] as a feedback or compensatory mechanism has been reported before in other studies. These data show that the mechanisms of resolution of inflammation were still taking place in the brains of aged mice long term after SI. The same challenge was not enough to upregulate cytokines in the brains of young mice long term after SI, suggesting that aged mice had a deficitary resolution response to episodic inflammation.

Neuroinflammation and oxidative stress are key processes involved in the pathophysiology of innumerous brain diseases, constituting the mechanism by which tissue damage occur during disease and aging [51, 52]. The role of reactive oxygen species (ROS) in neurodegeneration and cognitive decline has been extensively demonstrated in human patients and experimental models of diseases [51, 53–56]. Here, we observed that aged mice presented increased expression of *Nox2*, the phagocytic NADPH oxidase highly expressed in microglia, after SI (Fig. 5a). Nox2-derived ROS have been associated to microglia activation and postoperative cognitive dysfunction in mice [57]. Accordingly, HNE-protein adducts were significantly increased in whole brain extracts of aged mice after SI, while the HNE-immunostaining in the hippocampus was also higher in the aged mice after SI, compared to young controls (Fig. 5). These data indicate that SI induced an oxidative damage in the brains of aged mice after SI that was absent in the young mice submitted to the same treatment. The expression of antioxidant enzymes was also measured and found decreased in the brains of aged mice after SI (Fig. 6), which explains the higher oxidative stress found in the brains of aged mice after SI. *Sod1* was especially downregulated in the brains of aged mice after SI, compared to young mice and aged controls (Fig. 6). This SI-related *Sod1* decrease leads to increased oxidative damage in the brains of aged mice after SI, which may explain the cognitive deficits presented by those animals. These changes in the brain redox balance were temporally associated with behavior alterations of aged mice long term after SI.

## Conclusions

**Fig. 7:**
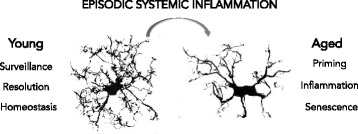
Overview of the effect of episodic systemic inflammation and aging on microglia. Young microglia have highly branched processes and are responsive to stimuli, contributing to tissue homeostasis. Aged microglia are dystrophic and resistant to regulation. Episodic systemic inflammation contributed to age-related microglia phenotype that was associated with age-related cognitive impairments

Taken together, our results show that aged mice present high susceptibility to cognitive impairments after episodic systemic inflammation that was associated to sustained oxidative stress and neuroinflammation. Our data show that aged microglia are primed and resistant to regulation, responding less effectively to inflammatory stimuli, a condition that might contribute to the poor cognitive performance of aged individuals that undergone episodic systemic inflammation (Fig. 7).

## Additional files

Additional file 1: Figure S1. Photomicrographs of senescence marker lipofuscin present in the aged brains only. Brain sections without any treatment incubated with mounting media for fluorescence (Vectashield), observed under fluorescence microscope show the tissue autofluorescence in the cortex of the aged mice. It is possible to observe the auto fluorescent aggregates in both the green (A) and the red (B) channels. Scale bar: 20 μm. (DOCX 3481 kb)

Additional file 2: Figure S2. Blood cytokines acutely after the first LPS injection. Blood cytokines were measured 4 h and 7 days after the first LPS injection. A) Blood IL-1β was significantly increased in aged mice 7 days after LPS. B) Blood IL-6 was less upregulated in aged mice 4 h after LPS injection. C) Blood IL-10 levels were not altered acutely. D) Blood IL-4 was significantly decreased in aged mice 7 days after LPS injection. Two-way ANOVA with Bonferroni posttest (*p < 0.05) and *t* test (#*p* < 0.05). (DOCX 192 kb)

Additional file 3: Figure S3. Blood cytokines long term after chronic low-grade systemic inflammation. Blood cytokines IL-1β (A), IL-6 (B), IL-4 (C), and IL-10 (D) were measured in young and aged mice naïve (control) and after chronic low-grade systemic inflammation (CSI). IL-4 levels remained significantly decreased in the blood of aged mice after CSI (*p < 0.05). (DOCX 96 kb)

## Abbreviations

HNE: Hydroxynonenal
Iba-1: Ionized calcium binding adaptor molecule 1
LPS: Lipopolysaccharide
ROS: Reactive oxygen species
SI: Episodic systemic inflammation
