# Supplementary material for: Age-related cognitive impairment is associated with long-term neuroinflammation and oxidative stress in a mouse model of episodic systemic inflammation
Source: J Neuroinflammation. 2018 Jan 30;15:28. doi: 10.1186/s12974-018-1059-y (PMC5791311; doi:10.1186/s12974-018-1059-y)
Supplement: Supplementary file 1 — Photomicrographs of senescence marker lipofuscin present in the aged brains only. Brain sections without any treatment incubated with mounting media for fluorescence (Vectashield), observed under fluorescence microscope show the tissue autofluorescence in the cortex of the aged mice. It is possible to observe the auto fluorescent aggregates in both the green (A) and the red (B) channels. Scale bar: 20 μm. (DOCX 3481 kb) [file 12974_2018_1059_MOESM1_ESM.docx]

**B**

**A**

**Additional file 1: Figure S1. Photomicrographs of senescence marker lipofuscin present in the aged brains only.** Brain sections without any treatment incubated with mounting media for fluorescence (Vectashield), observed under fluorescence microscope show the tissue autofluorescence in the cortex of the aged mice. It is possible to observe the auto fluorescent aggregates in both the green (A) and the red (B) channels. Scale bar: 20μm.
