# Supplementary material for: Age-related cognitive impairment is associated with long-term neuroinflammation and oxidative stress in a mouse model of episodic systemic inflammation
Source: J Neuroinflammation. 2018 Jan 30;15:28. doi: 10.1186/s12974-018-1059-y (PMC5791311; doi:10.1186/s12974-018-1059-y)
Supplement: Supplementary file 2 — Blood cytokines acutely after the first LPS injection. Blood cytokines were measured 4 h and 7 days after the first LPS injection. A) Blood IL-1β was significantly increased in aged mice 7 days after LPS. B) Blood IL-6 was less upregulated in aged mice 4 h after LPS injection. C) Blood IL-10 levels were not altered acutely. D) Blood IL-4 was significantly decreased in aged mice 7 days after LPS injection. Two-way ANOVA with Bonferroni posttest (*p < 0.05) and t test (#p < 0.05). (DOCX 192 kb) [file 12974_2018_1059_MOESM2_ESM.docx]

**Additional file 2: Figure S2. Blood cytokines acutely after the first LPS injection.** Blood cytokines were measured 4 hours and 7 days after de first LPS injection. A) Blood IL-1β was significantly increased in Aged mice 7 days after LPS. B) Blood IL-6 was less upregulated in Aged mice 4 hours after LPS injection. C) Blood IL-10 levels were not altered acutely by LPS treatment. D) Blood IL-4 was significantly decreased in Aged mice 7 days after LPS injection. Two- way ANOVA with Bonferroni post test (*P<0.05) and *t* test (#p<0,05).
