# Supplementary material for: Age-related cognitive impairment is associated with long-term neuroinflammation and oxidative stress in a mouse model of episodic systemic inflammation
Source: J Neuroinflammation. 2018 Jan 30;15:28. doi: 10.1186/s12974-018-1059-y (PMC5791311; doi:10.1186/s12974-018-1059-y)
Supplement: Supplementary file 3 — Blood cytokines long term after chronic low-grade systemic inflammation. Blood cytokines IL-1β (A), IL-6 (B), IL-4 (C), and IL-10 (D) were measured in young and aged mice naïve (control) and after chronic low-grade systemic inflammation (CSI). IL-4 levels remained significantly decreased in the blood of aged mice after CSI (*p < 0.05). (DOCX 96 kb) [file 12974_2018_1059_MOESM3_ESM.docx]

**Additional file 3: Figure S3. Blood cytokines long term after chronic low- grade systemic inflammation**. Blood cytokines IL-1β (A), IL-6 (B), IL-4 (C) and IL-10 (D) were measured in Young and Aged mice naïve (Control) and after chronic low- grade systemic inflammation (CSI). IL-4 levels remained significantly decreased in the blood of aged mice after CSI (*P<0.05).
